# Supplementary material for: Depopulation of dense α-synuclein aggregates is associated with rescue of dopamine neuron dysfunction and death in a new Parkinson’s disease model
Source: Acta Neuropathol. 2019 May 31;138(4):575–95. doi: 10.1007/s00401-019-02023-x (PMC6778064; doi:10.1007/s00401-019-02023-x)
Supplement: Supplementary file 1 — Supplementary material 1 (PDF 7407 kb) [file 401_2019_2023_MOESM1_ESM.pdf]

Supplementary material –Online Resource 1

Depopulation of dense α-synuclein aggregates is associated with rescue of dopamine neuron dysfunction and death in a new Parkinson's disease model

Michal Wegrzynowicz et al.

Supplementary Materials and Methods

*d*STORM

Monomeric αSyn definition and characterization

In order to characterize the dimensions of monomeric α-Syn by *d*STORM, we plated/stripped a solution containing full-length recombinant human α-Syn on the same coverslips used for the brain slices and stained the solution following the same protocol and primary and secondary antibodies, used for the striatum slices. We imaged the recombinant proteins using *d*STORM, and analyzed the size (mean diameter size, Supplementary Fig. S8a, Online Resource 1) and density of each one of the recombinant proteins particles using density-based (dbscan) cluster analysis (Supplementary Fig. S4 and Supplementary Fig. S8, Online Resource 1). We first used imageJ to identify putative centers of mass for each recombinant protein by loading all *d*STORM images of the proteins (Supplementary Fig. S8b, Online Resource 1). We removed very high or very low frequencies, to easily locate local intensity maxima, then we performed a reverse Fourier transform and used a build-in function in the program of tracking local maxima using a threshold of signal to noise to assure the maxima and avoid artifacts (Supplementary Fig. S8b, Online Resource 1). The tracked local maxima were then used as a reference to compare the tracking of putative clusters by the dbscan (density based algorithms) as was used for the slices analysis. Several parameters of ε (size of area for search) and k (threshold number of neighbors) were tested and compared to the results obtained from the imageJ. Finally the most accurate set of parameters for the dbscan was found to be ε=50 nm and k=4 neighbors (Supplementary Fig. S8b, Online Resource 1). Based on analysis of 870 putative recombinant proteins in 15 different images, the final analysis indicated that the size of a single monomeric

recombinant protein could correspond to a median size of 20.6 nm including a median number of 7 fluorophores per protein (Supplementary Fig. S8a, Online Resource 1).

Analysis of the non-clustered αSyn *population following anle138b treatment*

Using the definition and parameters of the monomeric recombinant αSyn analysis as described above, we characterized the population of αSyn proteins that were not defined as aggregates (non-clustered) in the mice treated with anle138b. The goal was to try to explore what the characteristics of this population are, whether the population is comprised mainly of monomers-like or higher orders of protein clusters/aggregates. When comparing anle138b-treated mice with placebo-treated animals, we found that the non-clustered population of proteins was substantially higher, 53.4% for anle138b-treated mice vs 23% for placebo-treated animals (Fig. 7d). We also noticed that among the population of non-clustered proteins there were aggregates with very low density, where the proteins in the aggregate seem dispersed (their density was defined as at least 10 times lower than the average density of aggregates from placebo-treated MI2 mice). In order to characterize more deeply these population of dispersed aggregates, we analyzed only this population of non-aggregated protein using the same parameters found for estimating recombinant monomeric αSyn as indicated above and we found that over 80% of the population is defined in a very similar way as a monomeric protein with a median diameter of 23 nm and median of 7 fluorophores per single cluster (protein). When looking at the overall distribution we can observe that the dispersed assemblies are composed of smaller assemblies with a mean diameter of 50-100 nm, that corresponds only to 10% of the total protein clusters (Supplementary Fig. S8c, Online Resource 1) which were surrounded or bridged to each other by what we had estimated as monomeric αSyn (around 85% of the total clusters, Supplementary

Fig. S8c, Online Resource 1). In Supplementary Fig. S8d, (Online Resource 1) a representative example of one of these dispersed assemblies is shown – with cores of smaller aggregates that are denser with a mean diameter size>50 nm and surrounded by many monomer-like species (mean diameter size=23 nm).

Supplementary Figures

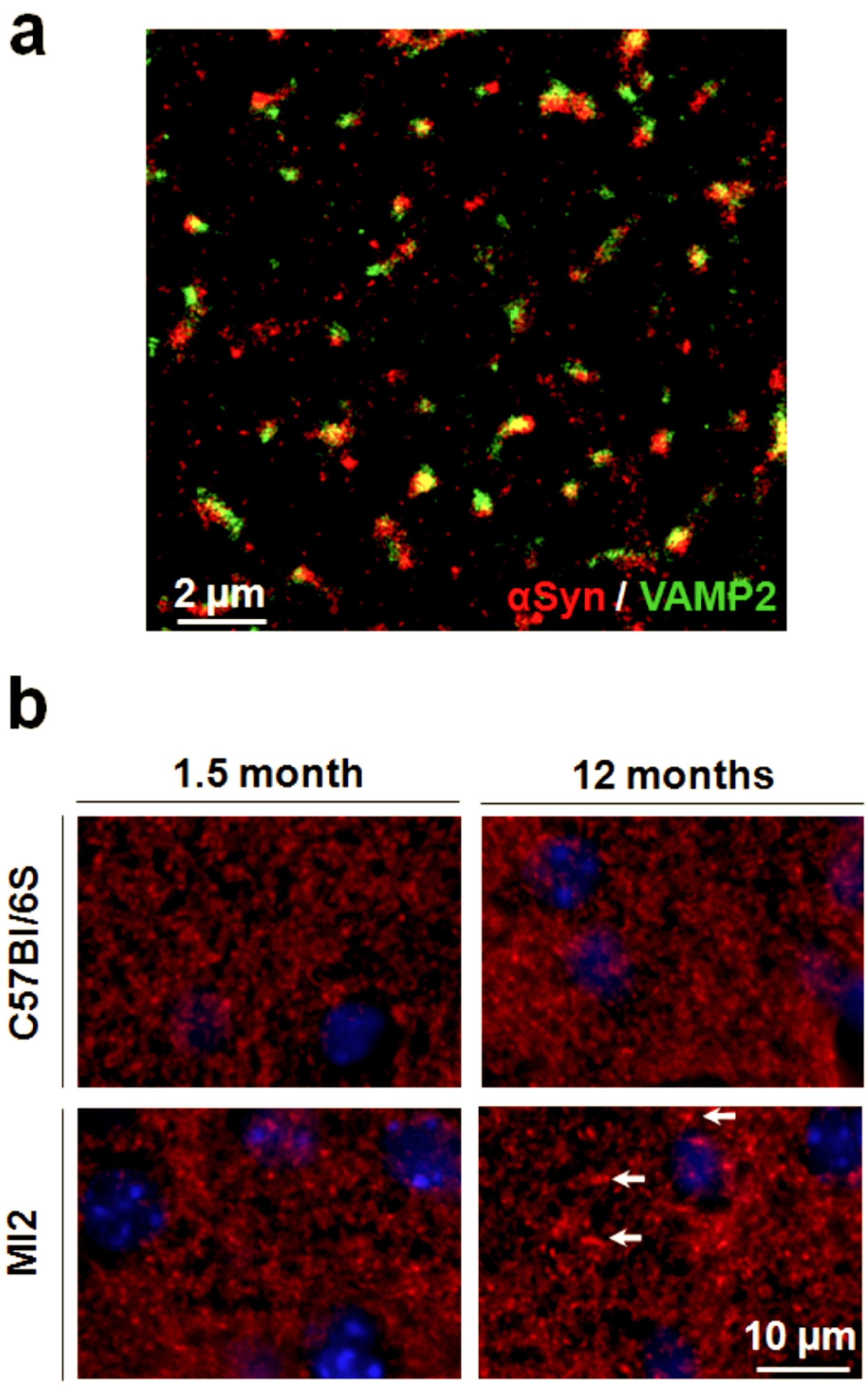

Supplementary Fig. S1 Co-localization of 1-120hαSYN and VAMP2 in striatal pre-synaptic compartment and VAMP2 redistribution. (**a**) Dual color *d*STORM image showing co-localization of 1-120hαSYN protein with synaptic marker, synaptobrevin (VAMP2), confirmed distribution of 1-120hαSYN protein in synaptic terminals in striatum of 6-month-old MI2 mice. (**b**) Redistribution of VAMP2 in striatum of MI2 mice. Distribution of VAMP2 in control C57Bl/6S mice is homogenous at both 1.5 and 12 months of age. MI2 mice exhibit progressive redistribution of striatal VAMP2. At 1.5 months of age, VAMP2 is distributed homogenously, similar to C57Bl/6S mice, at 12 months of age its distribution is visibly less homogenous, and numerous VAMP2-immunopositive clumps can be detected (arrows). Red-VAMP2 immunofluorescence, blue-nuclear staining with DAPI

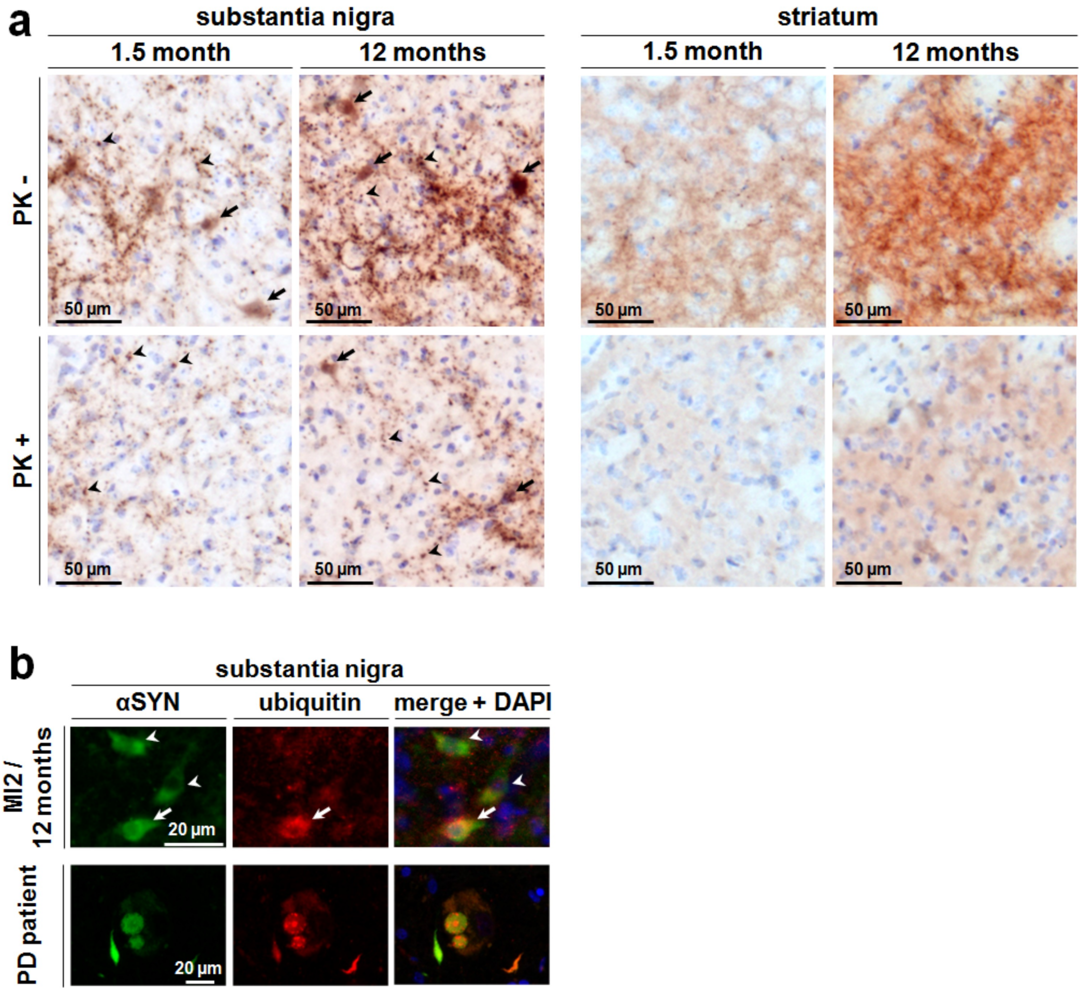

Supplementary Fig. S2 Proteinase K digestion of 1-120hαSYN aggregates in MI2 mice. (**a**) Proteinase K (PK) digestion assay. Nigral and striatal tissues from MI2 animals were treated with PK and residual 1-120hαSyn was detected with immunohistochemistry. In the SNpc, at 1.5 months of age, 1-120hαSyn immunoreactivity was strongly decreased following PK digestion (PK+), compared to non-treated tissue (PK-), however some residual PK-resistant αSYN was still detected in the form of punctate inclusions in some processes (arrowheads), but not in the cell bodies (arrows). At 12 months of age, following PK treatment more 1-120hαSyn immunoreactivity was detected in SNpc than at 1.5 months. Besides punctate inclusions in

processes (arrowheads), also sparse cells (arrows) contained PK-resistant 1-120hαSyn. In the striatum, at both 1.5 and 12 months of age, PK treatment completely abolished 1-120hαSyn immunoreactivity. (**b**) Ubiquitin staining in SNpc of MI2 mice. At 12 months of age, in SNpc, ubiquitin staining was found associated with 1-120hαSyn in some neurons (top panel, arrow) while other neurons contained only 1-120hαSyn (top panel, arrowheads). Specificity of ubiquitin staining was confirmed using section from PD patient SNpc, where ubiquitin antibody stained specifically Lewy bodies and Lewy neurites (bottom panel).

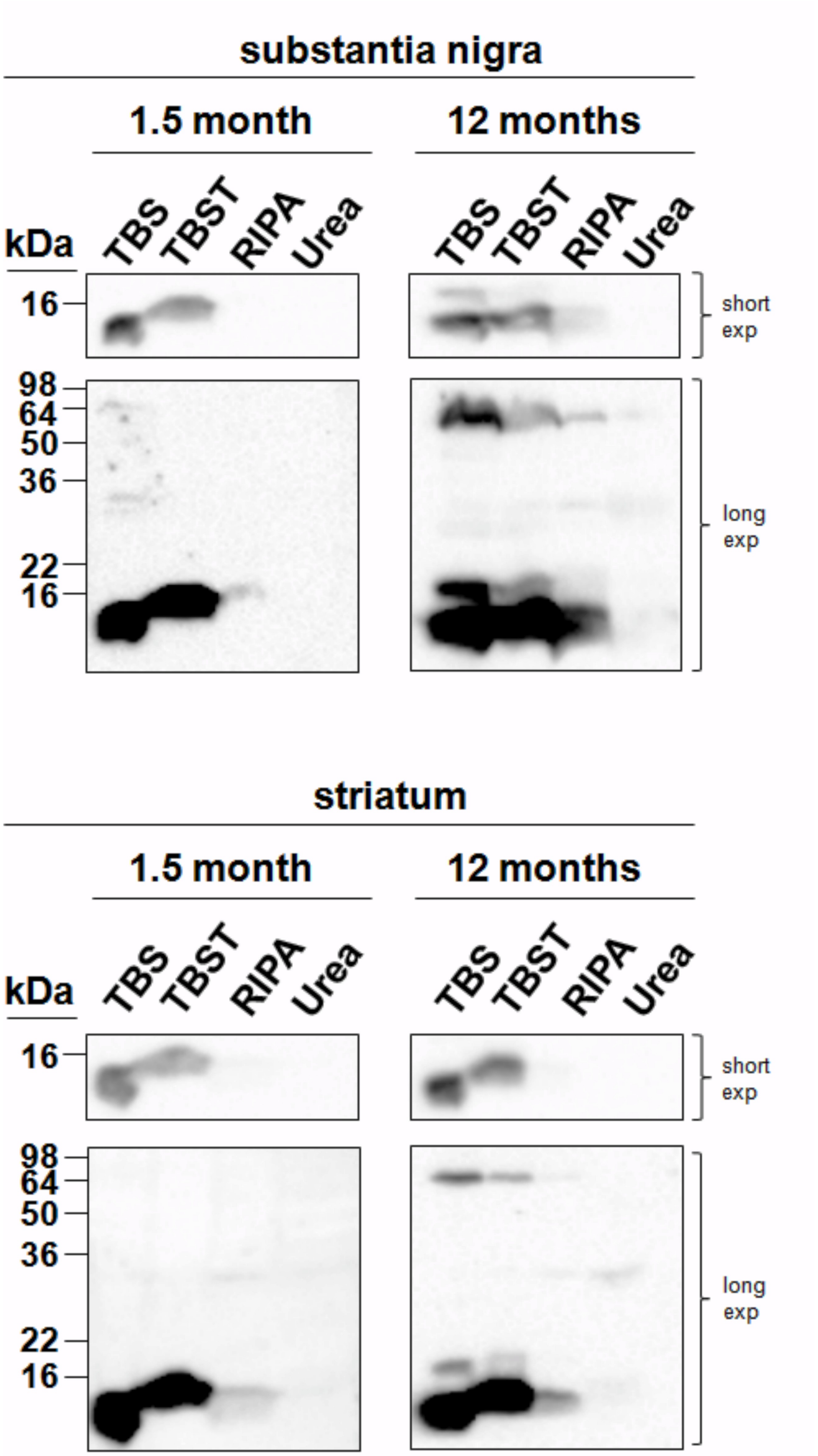

Supplementary Fig. S3 Progressive reduction of 1-120hαSyn solubility in MI2 mice. Nigral and striatal tissue from 1.5 and 12 month-old MI2 animals was subjected to sequential extraction of

proteins using buffers of gradually increasing strength. Increase in Triton-insoluble 1-120hαSyn (RIPA and urea fraction) was observed in both substantia nigra and striatum in 12 month-old mice compared to 1.5 month-old animals.

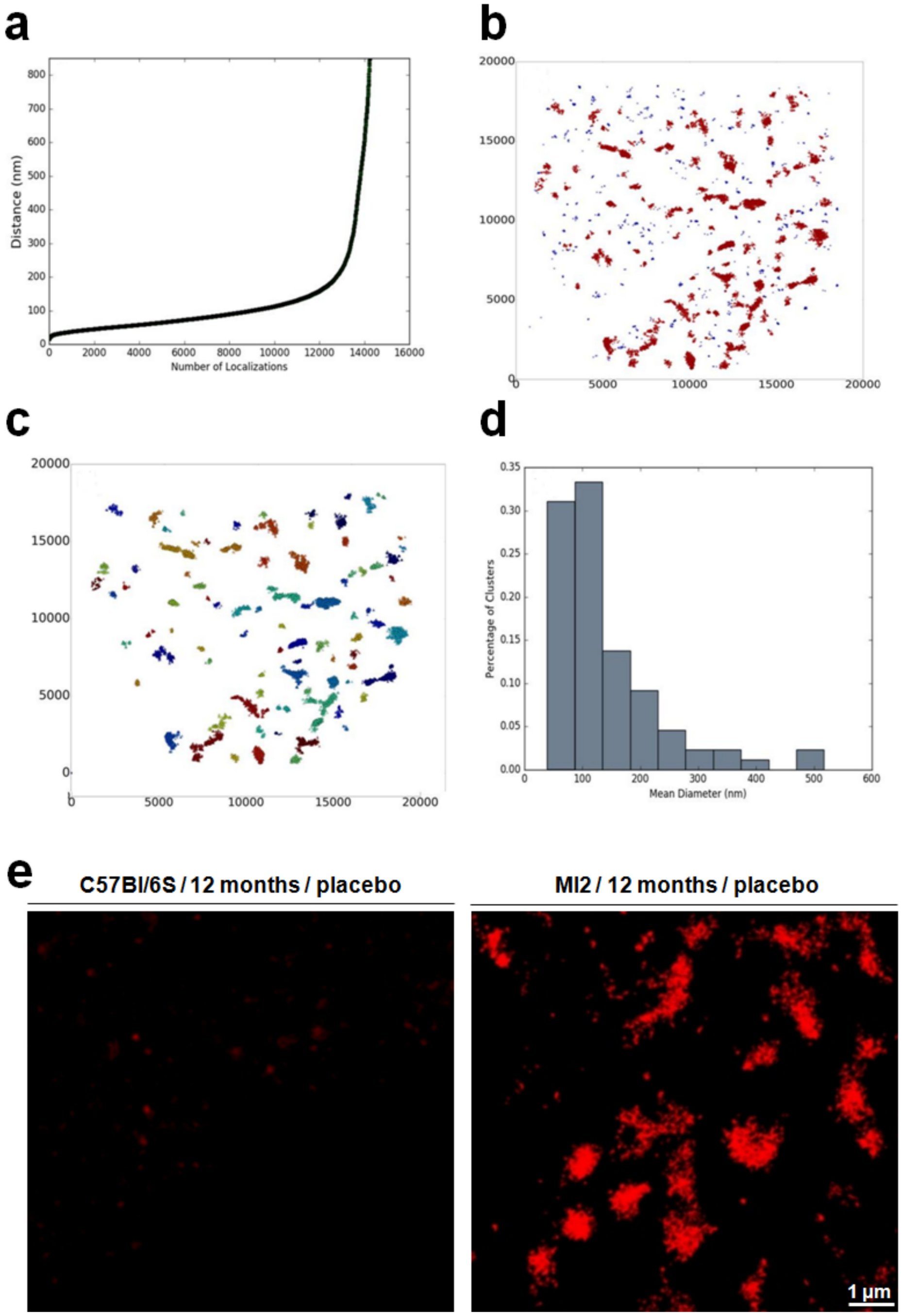

Supplementary Fig. S4 Analysis of *d*STORM data. (**a**) dbscan (density-based) cluster analysis was performed for the single localizations (predicted monomeric protein) in each image using the parameters of ε=200 and k=16 as determined by a set of k-dist measurement of nearest

neighbors analysis [1]. (**b**) Dividing protein localizations into clustered (aggregated; red) and non-clustered (non-aggregated / free (blue)) localizations (**c**) Different clusters / aggregates of αSYN are assigned with different colours, and for each aggregate the size, density, number of localization and shape is calculated. (**d**) Representative αSyn cluster size-distribution histogram in striatum of MI2 mice at 1.5 month of age. (**e**) Specificity of *d*STORM. Comparison between control αSyn-null C57Bl6/S mice (left: placebo-treated animals from anle138b experiment) and MI2 (right: placebo-treated MI2 animals) shows high specificity for αSyn *d*STORM. Non-specific background signal in C57Bl6/S mice striatum is easily distinguishable from the specific αSyn signal in MI2 animals.

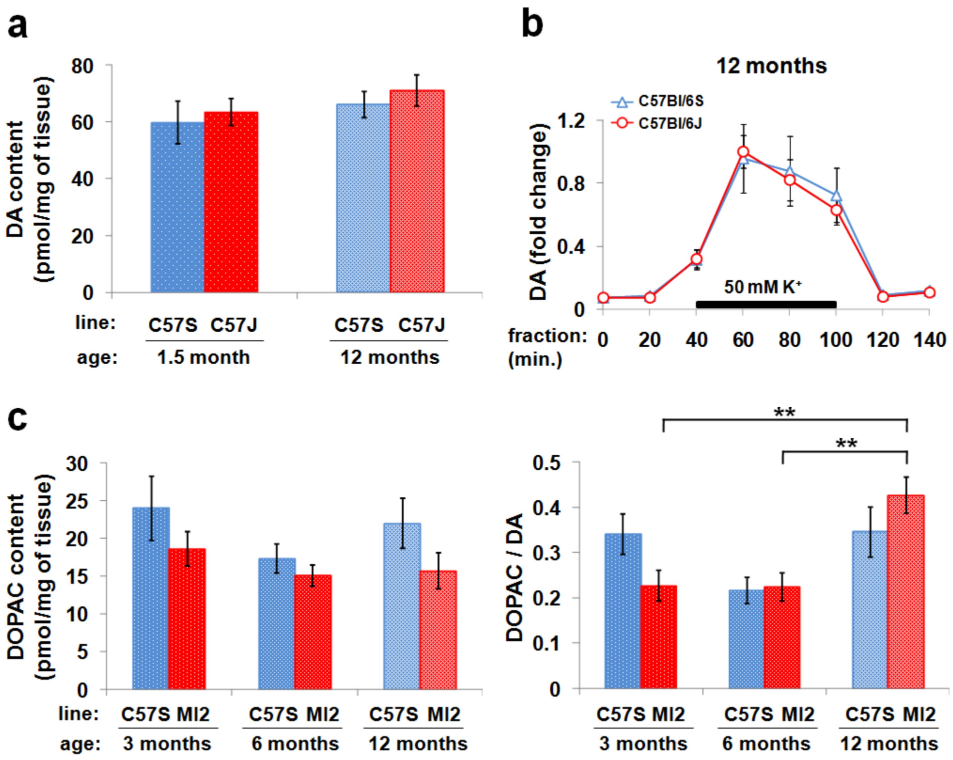

Supplementary Fig. S5 Comparison of striatal dopamine in αSyn-null C57Bl/6S control mice and endogenous αSyn-positive C57Bl/6J mice. (**a**) Total content of DA in striatal tissue was similar between C57Bl/6S and C57Bl/6J mice at 1.5 and 12 months of age. (**b**) No difference in DA release, measured by microdialysis, was found in the striata of C57Bl/6S and C57Bl/6J mice at 12 months of age as we have also shown previously [2]. (**c**) Analysis of DOPAC, a metabolite of dopamine degradation in the striatum of MI2 mice. DOPAC was measured in striatal lysates of MI2 and C57Bl/6S mice at 3, 6 and 12 months of age (mean ± SEM, n=5-8 mice per group; same samples as those used for DA measurements (Fig. 4a)). A main effect of genotype was identified by two-way ANOVA ($F(1,31)=4.610$; $p=0.04$), showing reduced levels of DOPAC over time in MI2 mice, however multiple comparisons with Bonferroni correction showed no difference between MI2 and C57Bl/6S mice at individual time points. For DOPAC/DA ratio, a main effect of age was identified by two-way ANOVA ($F(2,31)=8.825$; $p=0.001$), and multiple

comparisons with Bonferroni correction revealed statistically significant differences between 12 month-old MI2 mice and 3 month-old MI2 mice (**p=0.002) and between 12 month-old MI2 mice and 6 month-old MI2 mice (**p=0.003).

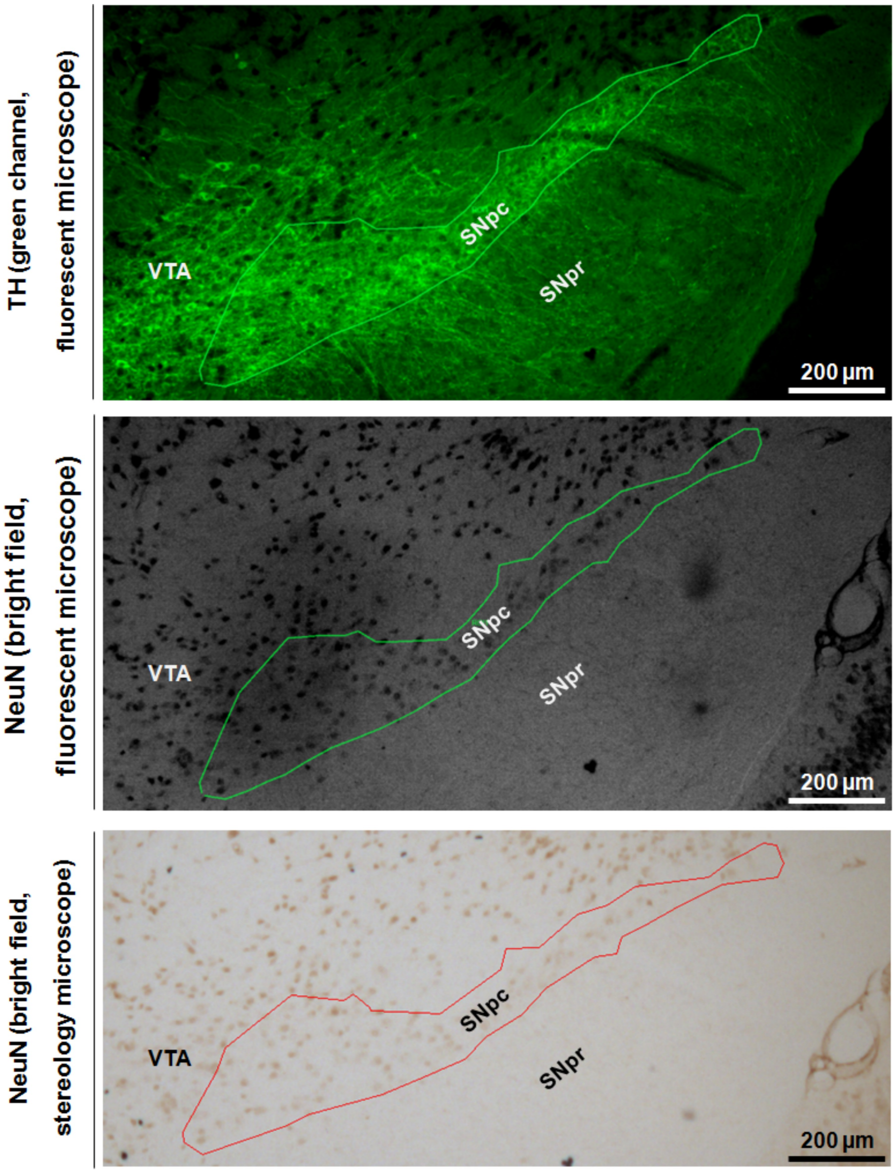

Supplementary Fig. S6 Representative image of combined peroxidase-based immunostaining of NeuN and immunofluorescence staining of TH for stereological counting of nigral NeuN-positive neurons. The double-stained sections were first analyzed using epifluorescent microscope. SNpc was contoured according to TH immunofluorescence in the green channel (upper panel), and the contour was superposed on the bright field image of NeuN immunohistochemistry (middle panel). Slides were then transferred to a stereology microscope and the contour of SNpc was precisely reconstructed in the bright field image in Stereo Investigator (bottom panel) for stereological counting of NeuN-positive nigral neurons. SNpc = substantia nigra pars compacta, SNpr = substantia nigra pars reticulata, VTA = ventral tegmental area.

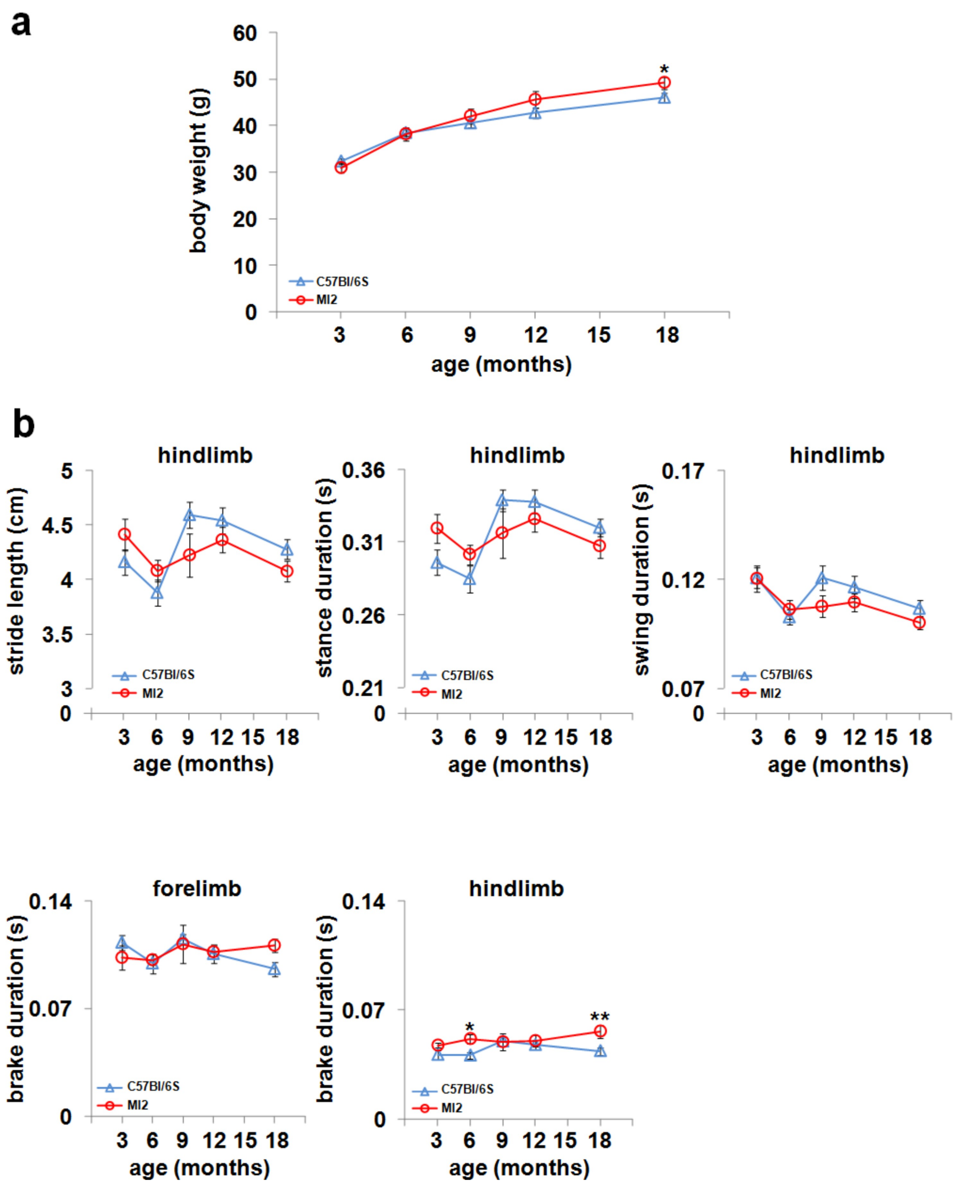

Supplementary Fig. S7 Comparison of body weight between MI2 and C57Bl/6S mice, and further analysis of gait pattern of MI2 mice. (**a**) Body weight of male MI2 and C57Bl/6S mice, used in DigiGait assay, was measured between 3 and 18 months of age (mean ± SEM, n=9-17 mice per group). A main effect of age was identified by two-way ANOVA ($F(4,99)=52.684$; $p<0.001$), and pairwise comparisons with Bonferroni correction between MI2 and C57Bl/6S

mice revealed statistically significant differences at 18 months of age (*p=0.025). (**b**) Further analysis of gait pattern of MI2 mice included hindlimb stride length, hindlimb stance and swing duration, and forelimb and hindlimb brake duration (mean ± SEM, n=9-17 mice per group). Two-way ANOVA identified a main effect of age on hindlimb stride length ($F(4,99)=4.638$; $p=0.002$), on hindlimb stance duration ($F(4,99)=5.044$; $p=0.001$), and on hindlimb swing duration ($F(4,99)=5.501$; $p<0.001$), and a main effect of genotype on hindlimb brake duration ($F(1,99)=7.524$; $p=0.007$). Statistically significant differences were identified in hindlimb brake duration at 6 months (*p=0.049) and at 18 months (**p=0.002) by pairwise comparisons with Bonferroni correction between MI2 and C57Bl/6S mice.

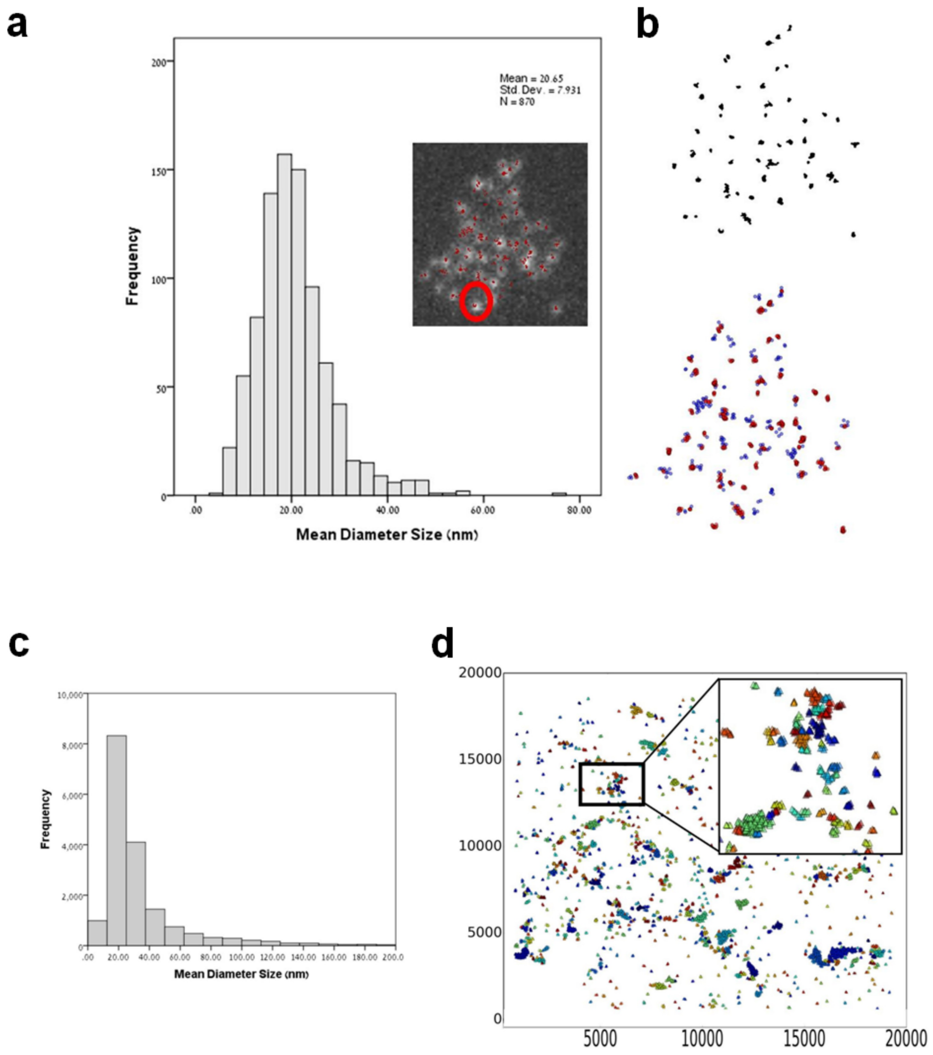

Supplementary Fig. S8 Identification of monomeric 1-120hαSyn in striatum of anle138b-treated MI2 mice. (**a**) Size distribution for monomeric recombinant full-length hαSyn. Putative single full-length hαSyn protein was imaged by *d*STORM and analysed using the dbscan analysis. The mean diameter size of a single hαSyn was found to be 20.65 nm. Inset: wide field image of recombinant hαSyn, with superposed fluorophore coordinates as imaged by *d*STORM (indicated in red). An example of a putative single hαSyn is marked by a red circle. (**b**) The parameters for a single hαSyn protein were detected using a combination of imageJ analysis for

finding centers of mass of proteins and dbscan analysis, both tools were combined to verify the definition of a single protein. (**c**) Size distribution of the non-clustered 1-120hαSyn population in striatum of anle138b-treated MI2 mice. 85% of 1-120hαSyn is in entities that would correspond to what we have defined as monomers (mean diameters size of 23 nm and median of ~7 fluorophores per cluster, similarly to the definition of the recombinant single protein. (**d**) A representative image of the non-clustered population composed of smaller low density dispersed aggregates and large population of monomers between them. Inset: representative dispersed aggregate, composed mainly of monomers (indicated in different colors).

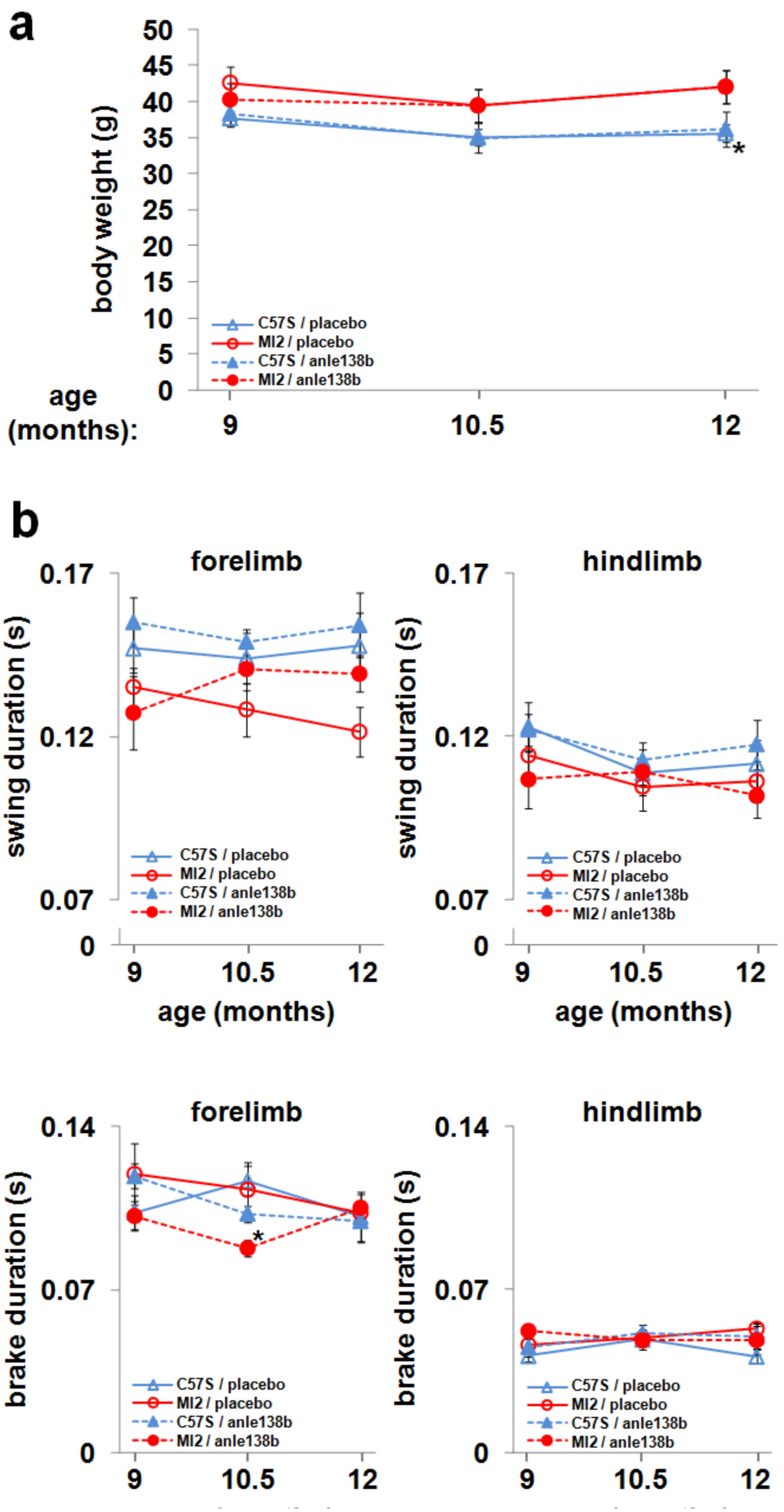
a
body weight (g)
age (months):
C57S / placebo
MI2 / placebo
C57S / anle138b
MI2 / anle138b
b
forelimb
hindlimb
swing duration (s)
brake duration (s)
age (months)

Supplementary Fig. S9 Comparison of body weight between placebo- and anle138b-treated MI2 and C57Bl/6S mice, and further analysis of anle138b effect on gait pattern in MI2 mice. (**a**) Body weight of placebo- and anle138b-treated MI2 and C57Bl/6S mice was measured at 9, 10.5 and 12 months of age (mean ± SEM, n=7-12 mice per group; males and females). Repeated measure ANOVA identified an effect of age, as a within-subject factor, on the body weight of animals over the course of the experiment ($F(2,72)=29.938$; $p<0.001$). Two-way ANOVA performed at individual time points identified an effect of genotype at 12 months of age ($F(1,36)=4.401$; $p=0.043$), and multiple comparisons with Bonferroni correction revealed statistically significant differences between placebo-treated C57Bl/6S and MI2 mice at 12 months of age (*p=0.038). (**b**) Further analysis of effect of anle138b on gait pattern in MI2 mice included forelimb and hindlimb swing and brake duration (mean ± SEM, n=6-12 mice per group; males and females). Three-way ANOVA revealed a main effect of genotype on forelimb ($F(1,96)=13.634$; $p<0.001$) and hindlimb ($F(1,96)=4.770$; $p=0.031$) swing duration. Two-way ANOVA performed at individual time points identified, at 10.5 months of age, an effect of treatment on forelimb brake duration ($F(1,33)=10.061$; $p=0.003$), and, at 12 months of age, an effect of genotype on forelimb swing duration ($F(1,27)=5.558$; $p=0.026$). Multiple comparisons with Bonferroni correction revealed statistically significant differences at 10.5 months of age between anle138b- and placebo-treated MI2 mice in forelimb brake duration (*p=0.013).

Detailed statistical evaluation of the results shown in the Figures in the main text

Fig. 1b One-way ANOVA revealed a main effect on 1-120hαSYN normalized to either β-actin or TH ($F(2,6)=99.813$, $p<0.001$, and $F(2,6)=22.97$, $p=0.002$, respectively). Multiple comparisons with Bonferroni correction revealed statistically significant differences between SN and OB ($p<0.001$ for αSYN/β-act, and $p=0.002$ for αSyn/TH) and between Str and OB ($p<0.001$ for αSyn/β-act and $p=0.005$ for αSyn/TH)

Fig. 2c In SN, one-way ANOVA revealed a main effect of age on αSyn/β-actin ($F(2,6)=10.540$, $p=0.011$) and αSyn/TH ($F(2,6)=6.416$, $p=0.032$). Multiple comparisons with Bonferroni correction revealed statistically significant difference between 1.5 and 12 months ($p=0.019$) and between 6 and 12 months ($p=0.026$) for αSyn/β-actin, and between 6 and 12 months ($p=0.042$) for αSyn/TH. In striatum, one-way ANOVA revealed a main effect of age on αSyn/β-actin ($F(2,6)=6.338$, $p=0.033$) and αSyn/TH ($F(2,6)=7.431$, $p=0.024$). Significant differences were present for αSyn/β-actin ($p=0.040$), and αSyn/TH ($p=0.026$) between 1.5 and 6 months

Fig. 3b A main effect of age on the number of aggregates (clusters) was identified ($F(2,6)=7.567$, $p=0.023$) by one-way ANOVA, and multiple comparisons with Bonferroni correction revealed statistically significant difference between 1.5 and 12 months of age ($p=0.03$). No differences in the aggregate median size or number of localizations per cluster were found

Fig. 3c Analysis of cluster size distribution has shown statistically significant increase in the number of medium-size aggregates - one way ANOVA revealed a main effect of age on the abundance of 100-300 nm ($F(2,6)=9.784$, $p=0.013$) and 300-500 nm ($F(2,6)=7.668$, $p=0.022$) clusters, and an increase between 1.5 and 12 months of age in these populations of clusters was revealed by multiple comparisons with Bonferroni correction ($p=0.014$, $p=0.024$ for 100-300 nm and 300-500 nm clusters, respectively). Direct comparison with t-test revealed also a statistically significant difference between 1.5 and 12 month-old animals for 20-100 nm clusters ($p=0.013$)

Fig. 4a Two-way ANOVA identified a main effect of age ($F(2,31)=6.566$, $p=0.004$) and a statistically significant interaction between genotype and age ($F(2,31)=3.849$, $p=0.032$). Multiple comparisons with Bonferroni correction revealed significant differences between C57Bl/6S and MI2 animals at 12 months of age ($p=0.03$) and between 3 and 12 month-old MI2 mice ($p<0.001$) and 6 and 12 month-old MI2 mice ($p=0.019$)

Fig. 4b At 3 months of age no difference between MI2 and C57Bl/6S mice was observed. At 6 months, interaction between genotype and sample time was identified by two-way mixed ANOVA ($F(6,48)=3.470$, $p=0.006$). In MI2 mice, following K^+ stimulation, DA release was reduced compared to C57Bl/6S (t-test for individual sampling time points: $p=0.038$ at 60 min., $p=0.009$ at 100 min.). In older mice this deficit was more prominent (at 9 months - genotype x sample time interaction, $F(6,54)=8.965$, $p<0.001$; t-test, $p=0.013$ at 60 min, $p=0.015$ at 80 min, $p=0.015$ at 100 min; at 12 months - genotype x sample time interaction, $F(6,30)=17.322$, $p<0.001$; t-test, $p=0.011$ at 60 min, $p=0.008$ at 80 min, $p=0.028$ at 100 min)

Fig. 5b A main effect of genotype (F(1,24)=24.981, p<0.001) was identified by two-way ANOVA, and a significant reduction of TH+ neurons in MI2 mice compared to C57Bl/6S controls at 12 (p=0.018) and 20 (p<0.001) months of age, and between 9- and 20 months-old MI2 animals (p=0.015) were found using multiple comparisons with Bonferroni correction

Fig. 5e A main effect of genotype (F(1,18)=9.967, p<0.005) was identified by two-way ANOVA, and significant differences between C57Bl/6S and MI2 mice at 12 months of age (p=0.003), and between 9 and 12 month-old MI2 animals (p=0.017) were found using multiple comparisons with Bonferroni correction

Fig. 6a A main effect of age on the rotarod performance was identified by two-way ANOVA (F(3,108)=7.299, p<0.001). Multiple comparisons with Bonferroni correction revealed statistically significant difference between 6 and 20 months ($^{\#\#\#}$p<0.001) and 12 and 20 months (‡‡‡p<0.001) in MI2 mice, and between MI2 and C57Bl/6S animals at 20 months (p=0.012)

Fig. 6b No statistically significant differences between MI2 and C57Bl/6S mice were identified using 25 mm rod (t-tests, p=0.082 and p=0.103, orientation time and transit time, respectively). No difference in orientation time between experimental groups was found using 15 mm rod (t-test, p=0.096), but there was a statistically significant difference between MI2 and C57Bl/6S animals in transit time on 15 mm rod (p=0.028, t-test)

Fig. 6c Two-way ANOVA identified a main effect of age on forelimb stance (F(4,99)=3.083; p=0.019) and swing (F(4,99)=3.277; p=0.014) duration, and on hindlimb propulsion duration (F(4,99)=4.057; p=0.004), a main effect of genotype on forelimb stride length (F(1,99)=13.222; p<0.001), and stance (F(1,99)=8.721; p=0.004), swing (F(1,99)=13.127; p<0.001) and propulsion (F(1,99)=11.160; p=0.001) duration, and two-way interaction between age and genotype for forelimb propulsion duration (F(4,99)=3.599; p=0.009). Statistically significant differences were identified by pairwise comparisons with Bonferroni correction between MI2 and C57Bl/6S mice for forelimb stride length at 9 (p=0.01), 12 (p=0.012) and 18 (p=0.009) months, for forelimb stance duration at 9 (p=0.015), 12 (p=0.027) and 18 (p=0.029) months of age, for forelimb swing duration at 9 (p=0.044), 12 (p=0.009) and 18 (p=0.007) months of age, for forelimb propulsion duration at 9 (p=0.021), 12 (p=0.014) and 18 (p<0.001) months of age and for hindlimb propulsion duration at 18 months of age (p=0.019).

Fig. 7d Statistically significant differences between anle138b- and placebo-treated MI2 mice were identified by t-test for inner cluster density (p=0.031) and percentage of non-clustered 1-120 hαSyn (p=0.005)

Fig. 7e Statistically significant differences between anle138b- and placebo-treated MI2 mice were identified by t-test for high molecular weight (p=0.005) and monomeric (p=0.009) 1-120hαSYN

Fig. 8a Three-way mixed ANOVA identified an effect of genotype (F(1,17)=10.733, p<0.001), effect of sampling time (F(6,102)=41.756, p<0.001), two-way genotype x sampling time

interaction ($F(6,102)=8.416$, $p<0.001$) and three-way interaction between sampling time, genotype and treatment ($F(6,102)=2.355$, $p=0.036$). Two-way ANOVA run for individual sampling time points identified a main effect of genotype at 60 min ($F(1,18)=10.468$, $p=0.005$), 80 min ($F(1,18)=12.126$, $p=0.003$) and 100 min ($F(1,18)=10.146$, $p=0.005$), and two-way genotype x treatment interaction at 80 min ($F(1,18)=7.537$, $p=0.013$). Multiple comparisons with Bonferroni correction revealed statistically significant differences between placebo-treated MI2 and C57Bl/6S mice at 60, 80 and 100 min ($p=0.004$, $p<0.001$, $p=0.003$, respectively) and between placebo- and anle138b-treated MI2 mice at 60, 80 and 100 min ($p=0.048$, $p=0.018$, $p=0.038$, respectively)

Fig. 8c A main effect of genotype was identified by two-way ANOVA ($F(1,8)=11.682$, $p=0.009$). Multiple comparisons with Bonferroni correction revealed statistically significant difference in number of nigral DA neurons between placebo-treated C57Bl/6S and MI2 mice ($p=0.005$), but not between anle138b-treated animals ($p=0.365$). There was also a significant difference between placebo- and anle138b-treated MI2 mice ($p=0.023$)

Fig. 9 Three-way ANOVA revealed a main effect of age on forelimb ($F(2,96)=4.926$; $p=0.009$) and hindlimb ($F(2,96)=6.006$; $p=0.003$) stride length, on forelimb ($F(2,96)=14.597$; $p<0.001$) and hindlimb ($F(2,96)=7.463$; $p=0.001$) stance duration, and on forelimb ($F(2,96)=5.627$; $p=0.005$), and hindlimb ($F(2,96)=9.626$; $p<0.001$) propulsion duration, a main effect of genotype on forelimb ($F(1,96)=18.426$; $p<0.001$) and hindlimb ($F(1,96)=21.025$; $p<0.001$) stride length, on forelimb ($F(1,96)=13.320$; $p<0.001$) and hindlimb ($F(1,96)=27.330$; $p<0.001$) stance duration, and on forelimb ($F(1,96)=7.467$; $p=0.007$) and hindlimb ($F(1,96)=34.908$; $p<0.001$) propulsion

duration, a main effect of treatment on hindlimb stance ($F(1,96)=6.819$; $p=0.01$) and propulsion ($F(1,96)=4.839$; $p=0.03$) duration, and a three-way age x genotype x treatment interaction for forelimb stance duration ($F(2,96)=3.502$; $p=0.034$). Two-way ANOVA, performed at individual time points, identified, at 10.5 months of age, an effect of genotype on forelimb ($F(1,33)=9.797$; $p=0.004$) and hindlimb ($F(1,33)=8.277$; $p=0.007$) stride length, on forelimb ($F(1,33)=12.016$; $p=0.001$) and hindlimb ($F(1,33)=14.831$; $p=0.001$) stance duration, and on forelimb ($F(1,33)=5.938$; $p=0.02$) and hindlimb ($F(1,33)=13.516$; $p=0.001$) propulsion duration, and an effect of treatment on hindlimb stance duration ($F(1,33)=4.640$; $p=0.039$) and on forelimb propulsion duration ($F(1,33)=4.545$; $p=0.041$), and, at 12 months of age, an effect of genotype on forelimb ($F(1,27)=9.574$; $p=0.005$) and hindlimb ($F(1,27)=11.534$; $p=0.002$) stride length, on forelimb ($F(1,27)=5.765$; $p=0.024$) and hindlimb ($F(1,27)=12.565$; $p=0.001$) stance duration, and on forelimb ($F(1,27)=4.711$; $p=0.039$), and hindlimb ($F(1,27)=15.111$; $p=0.001$) propulsion duration. Multiple comparisons with Bonferroni correction revealed, at 10.5 months of age, statistically significant differences between placebo-treated C57Bl/6S and MI2 mice in forelimb ($p=0.013$) and hindlimb ($p=0.016$) stride length, in forelimb ($p=0.008$) and hindlimb ($p=0.001$) stance duration, and in forelimb ($p=0.017$) and hindlimb ($p=0.001$) propulsion duration, and between placebo- and anle138b-treated MI2 mice in hindlimb stance duration ($p=0.027$) and in forelimb ($p=0.034$) and hindlimb ($p=0.026$) propulsion duration, and, at 12 months of age, statistically significant differences between placebo-treated C57Bl/6S and MI2 mice in forelimb ($p=0.007$) and hindlimb ($p=0.024$) stride length, in forelimb ($p=0.016$) and hindlimb (0.008) stance duration, and in hindlimb propulsion duration ($p=0.016$), between anle138b-treated C57Bl/6S and MI2 mice in hindlimb stride length ($p=0.022$) and stance duration ($p=0.044$) and

between placebo- and anle138b-treated MI2 mice in forelimb stride length (p=0.04) and stance duration (p=0.048).

REFERENCES

1. Bar-On D, Wolter S, van de Linde S, Heilemann M, Nudelman G, Nachliel E, Gutman M, Sauer M, Ashery U (2012) Super-resolution imaging reveals the internal architecture of nano-sized syntaxin clusters. J Biol Chem 287:27158-27167.
2. Garcia-Reitboeck P, Anichtchik O, Dalley JW, Ninkina N, Tofaris GK, Buchman VL, Spillantini MG (2013) Endogenous alpha-synuclein influences the number of dopaminergic neurons in mouse substantia nigra. Exp Neurol 248:541-545.
